# COGNITION OLDER ADULTS WITH PREDIABETES EXPERIENCE ACCELERATED NEUROCOGNITIVE DECLINE

**DOI:** 10.1093/geroni/igz038.2427

**Published:** 2019-11-08

**Authors:** Joyla Furlano, Lindsay Nagamatsu

**Affiliations:** Western University, London, Ontario, Canada

## Abstract

Type II diabetes (T2D) is associated with neurocognitive decline beyond normative aging, and thus older adults with T2D are at high risk for developing dementia. However, the extent to which similar deficits occur in prediabetic older adults is not well understood. While few studies have shown that prediabetic older adults experience some cognitive decline, further research is needed to determine the specific cognitive domains affected and the degree to which this decline occurs. Moreover, structural and functional brain changes that may occur with these deficits is currently unknown in this population. Therefore, the aim of this study was to assess cognitive function and brain health in prediabetic older adults. We conducted a cross-sectional analysis of older adults (aged 60-80) with prediabetes (FPG 6.1-7.0 mmol/L) and healthy aged-matched controls, examining 1) cognitive performance, 2) functional brain activation as measured by fMRI, and 3) structural measures such as volume of the hippocampus. Based on our cross-sectional analysis, prediabetic older adults show impaired cognition (e.g., memory), as well as decreased hippocampal volume and activation. Therefore, we conclude that older adults with prediabetes experience brain decline, and could benefit from lifestyle interventions to prevent or delay the onset of such decline.

